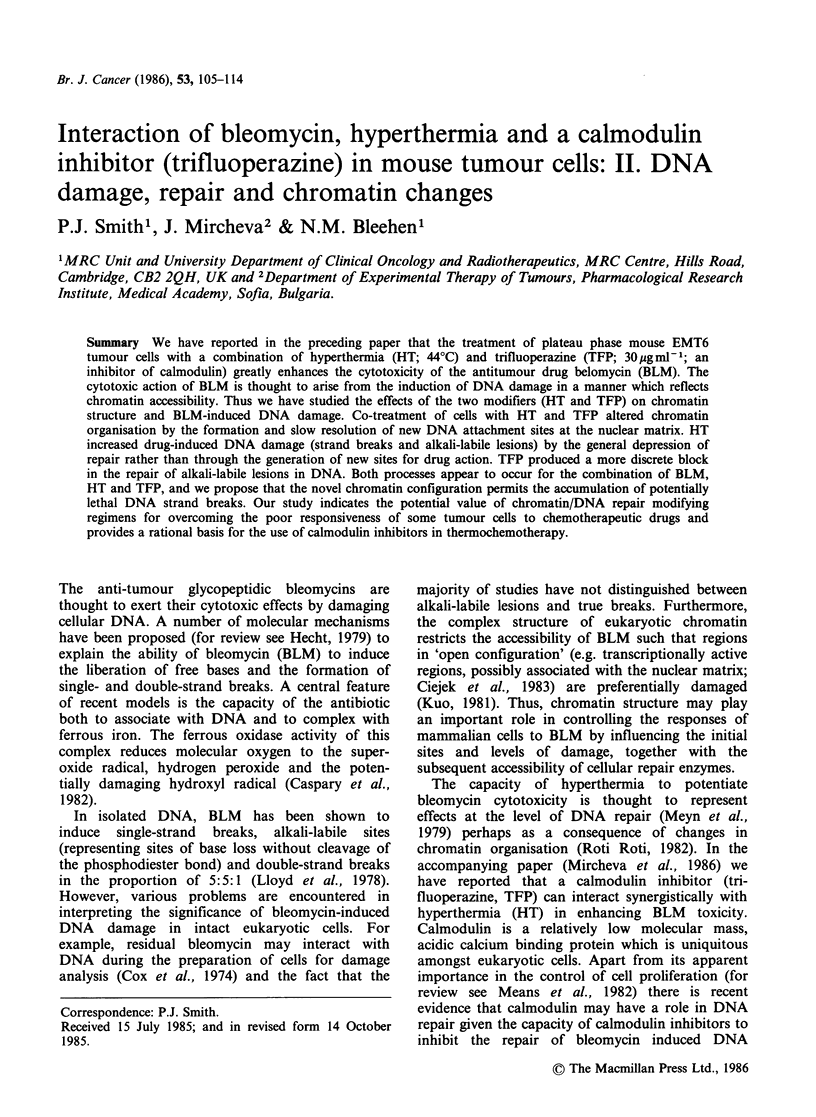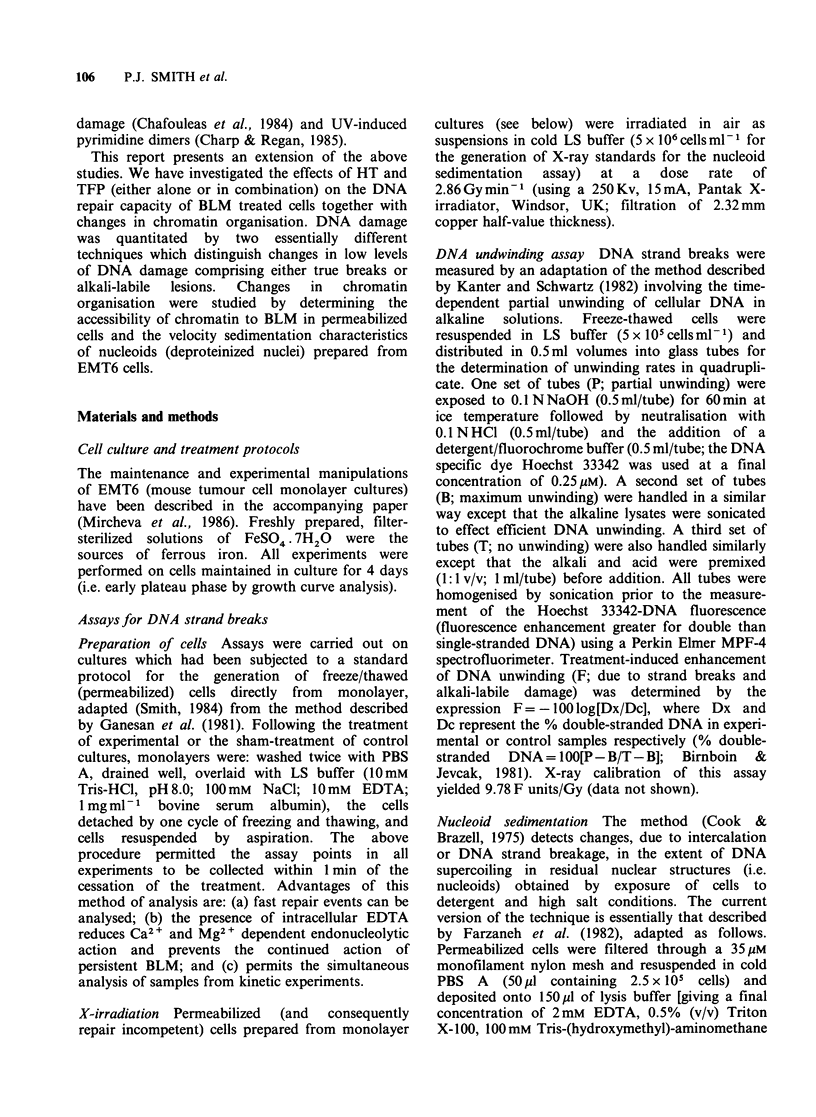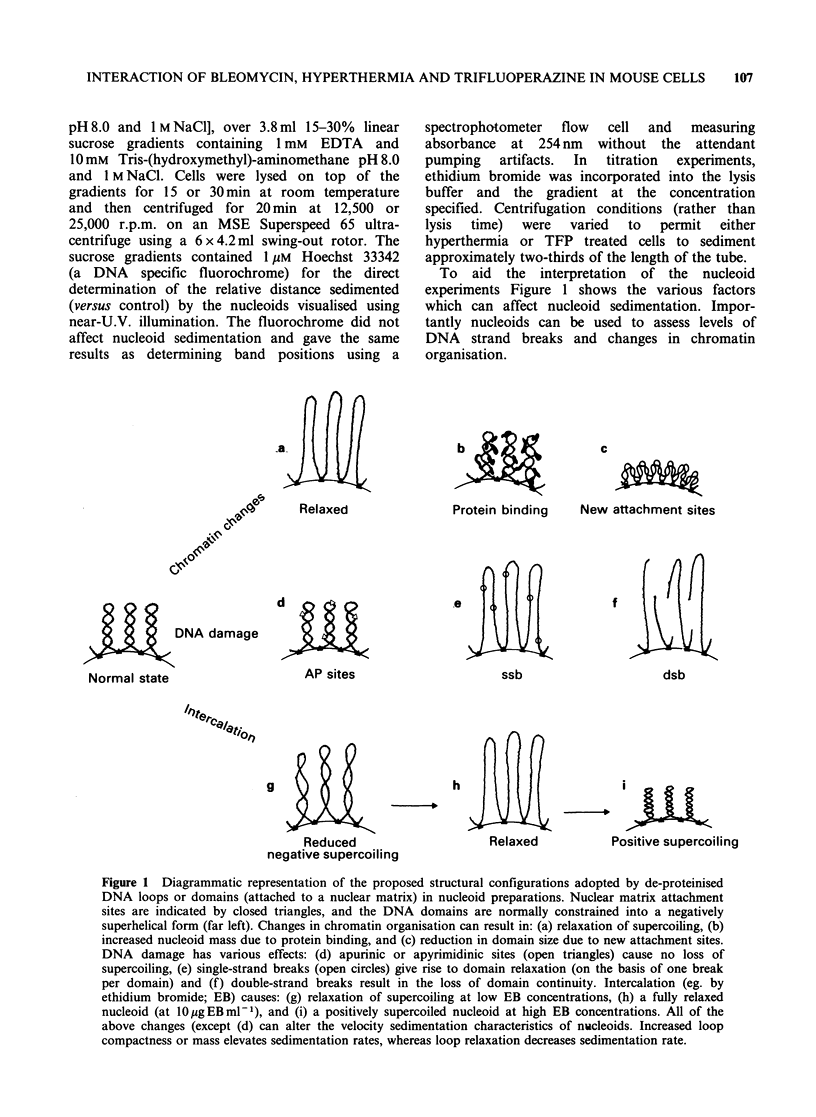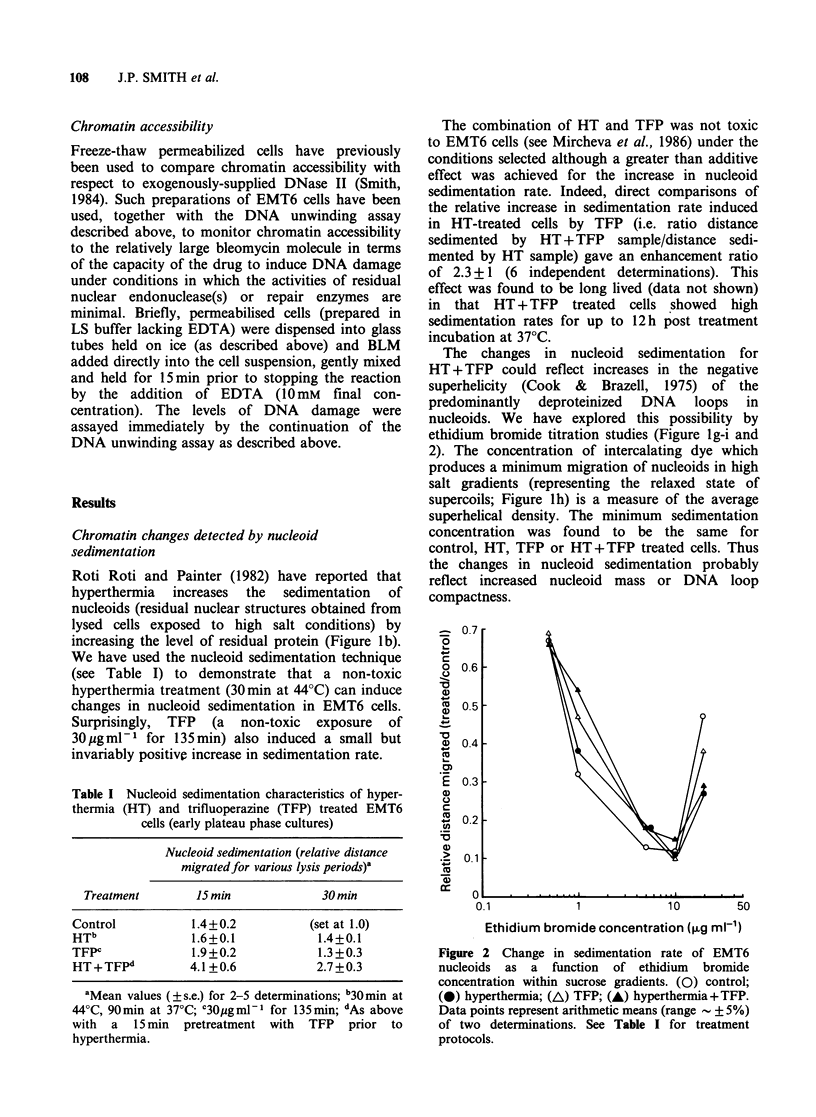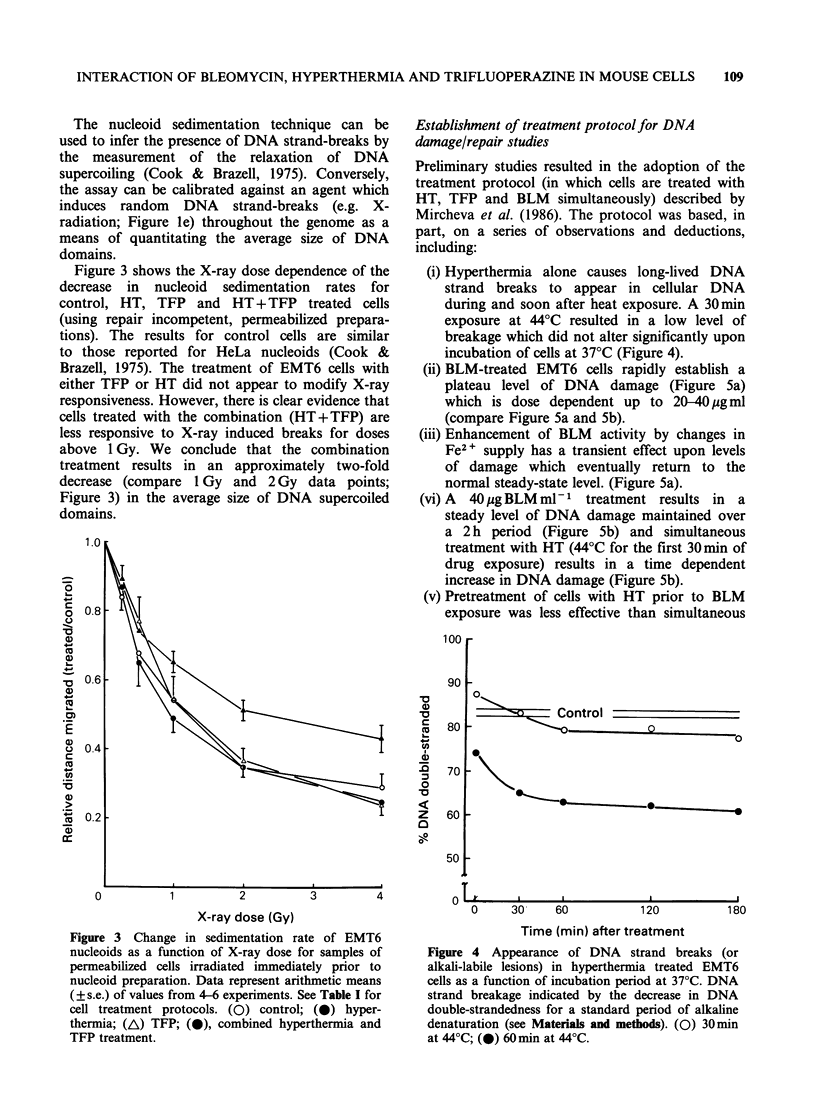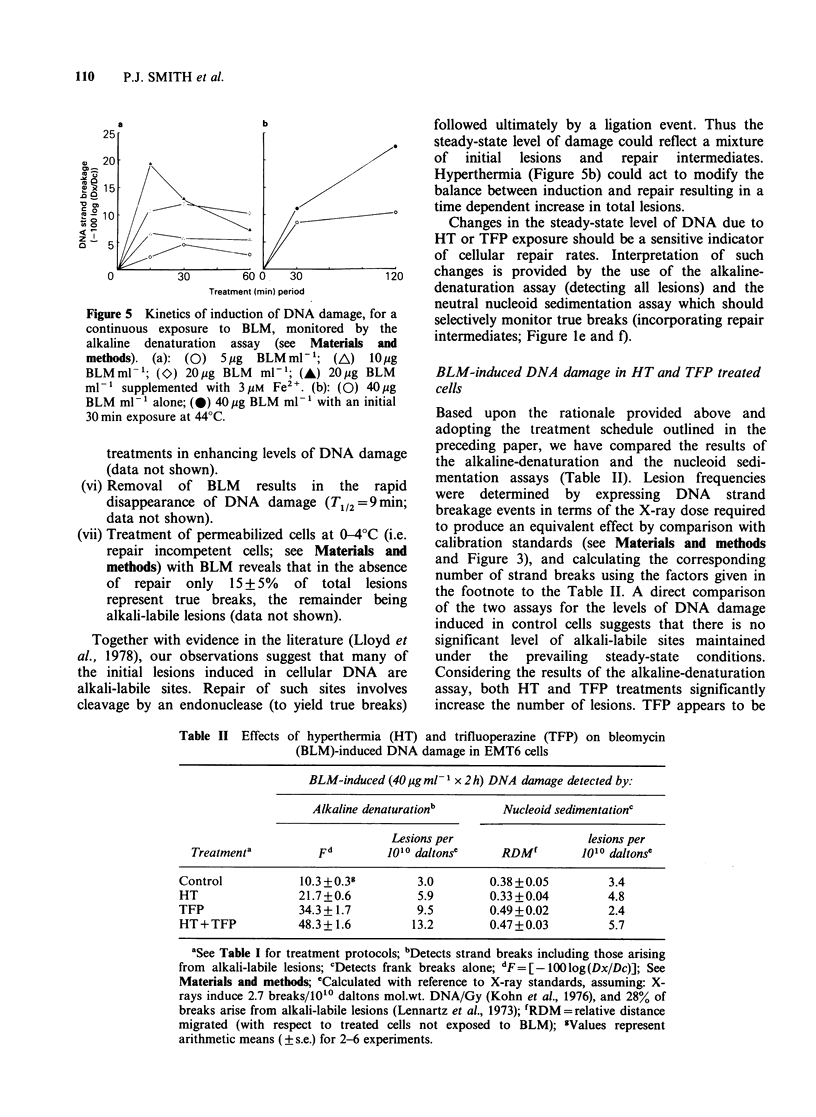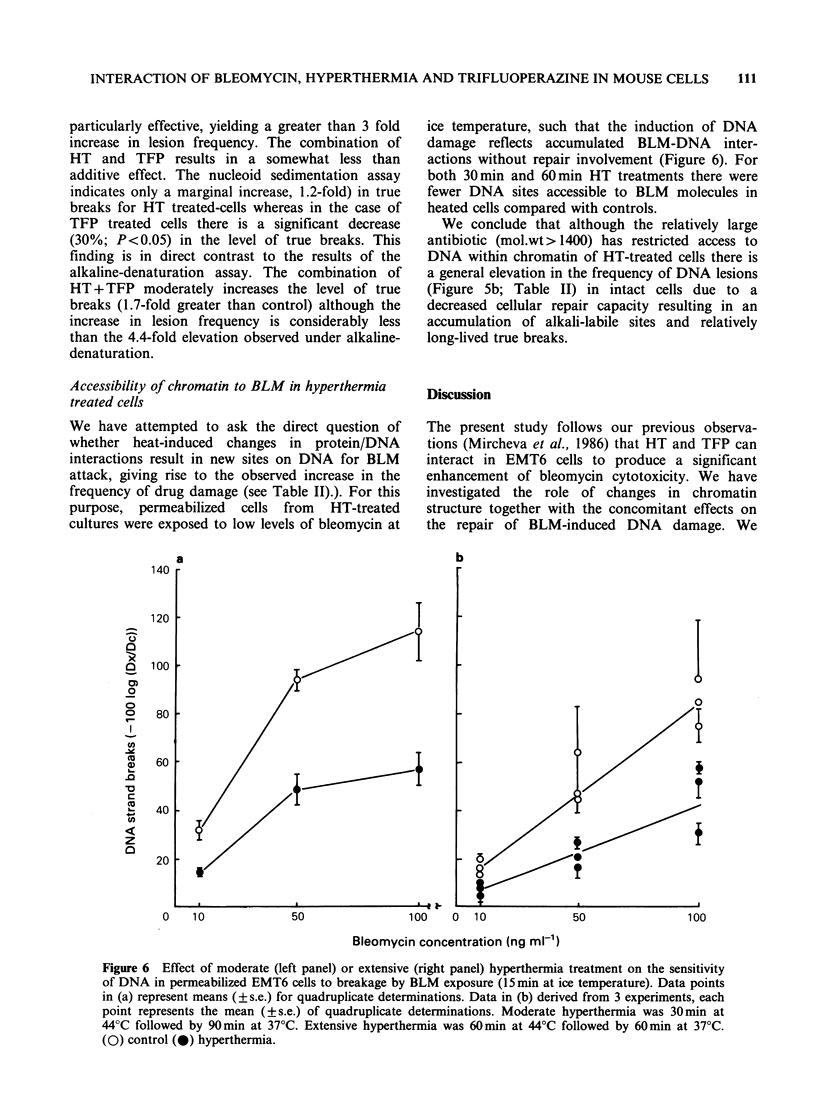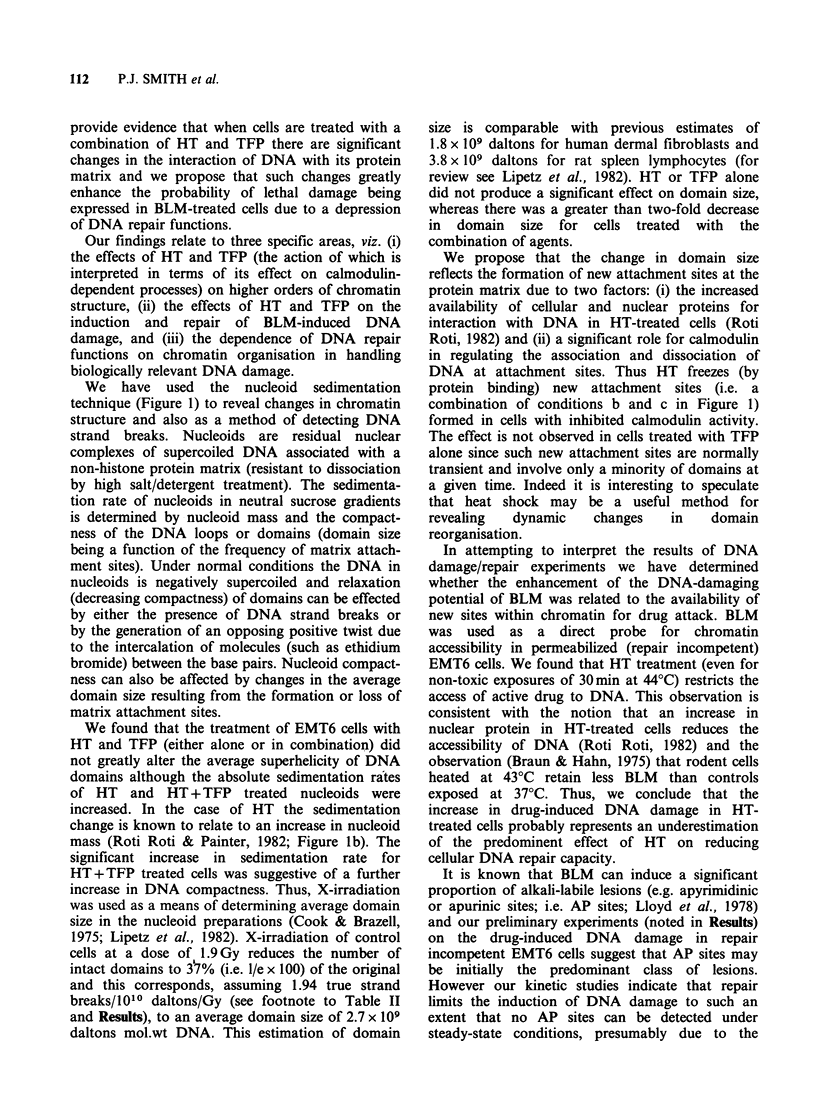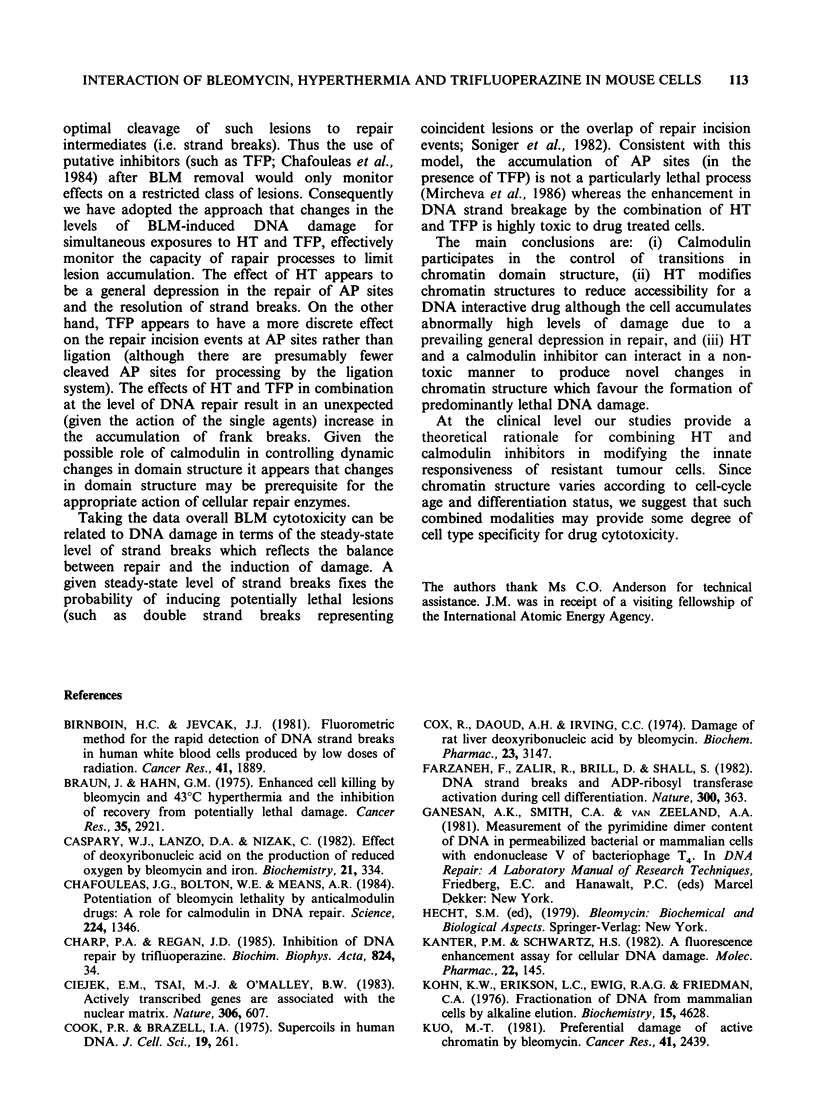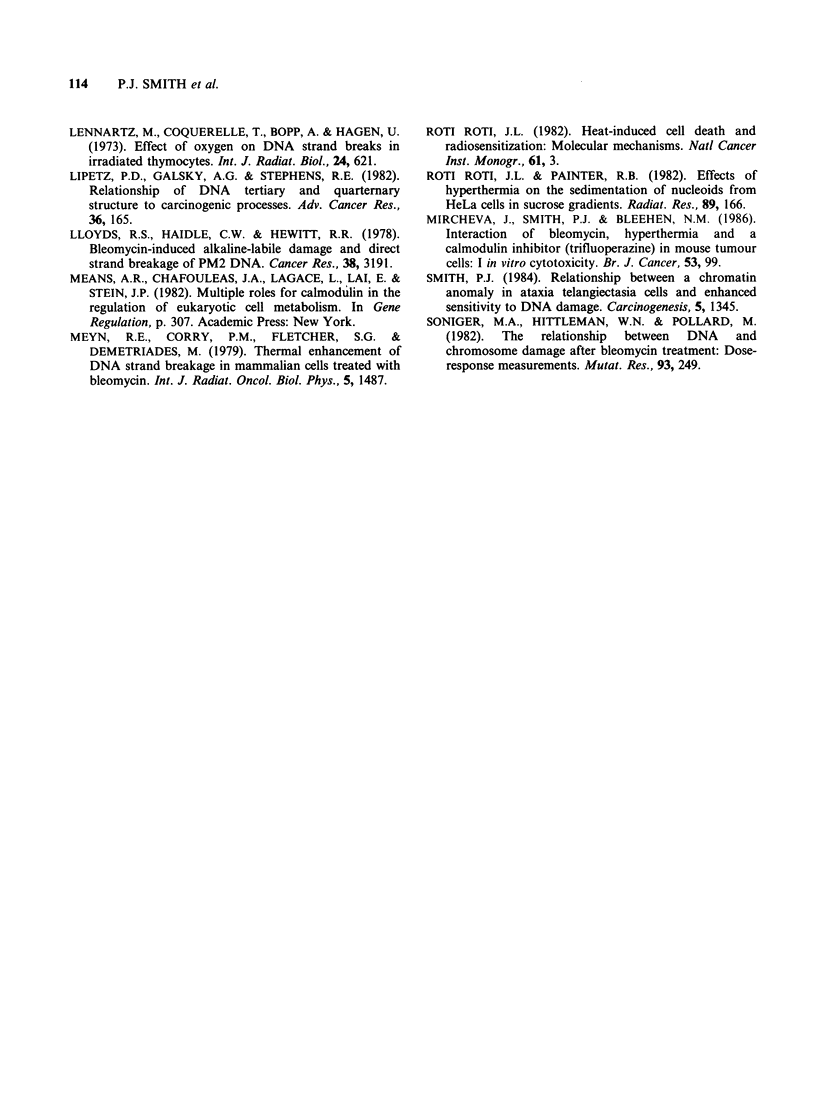# Interaction of bleomycin, hyperthermia and a calmodulin inhibitor (trifluoperazine) in mouse tumour cells: II. DNA damage, repair and chromatin changes.

**DOI:** 10.1038/bjc.1986.15

**Published:** 1986-01

**Authors:** P. J. Smith, J. Mircheva, N. M. Bleehen

## Abstract

We have reported in the preceding paper that the treatment of plateau phase mouse EMT6 tumour cells with a combination of hyperthermia (HT; 44 degrees C) and trifluoperazine (TFP; 30 micrograms ml-1; an inhibitor of calmodulin) greatly enhances the cytotoxicity of the antitumour drug belomycin (BLM). The cytotoxic action of BLM is thought to arise from the induction of DNA damage in a manner which reflects chromatin accessibility. Thus we have studied the effects of the two modifiers (HT and TFP) on chromatin structure and BLM-induced DNA damage. Co-treatment of cells with HT and TFP altered chromatin organisation by the formation and slow resolution of new DNA attachment sites at the nuclear matrix. HT increased drug-induced DNA damage (strand breaks and alkali-labile lesions) by the general depression of repair rather than through the generation of new sites for drug action. TFP produced a more discrete block in the repair of alkali-labile lesions in DNA. Both processes appear to occur for the combination of BLM, HT and TFP, and we propose that the novel chromatin configuration permits the accumulation of potentially lethal DNA strand breaks. Our study indicates the potential value of chromatin/DNA repair modifying regimens for overcoming the poor responsiveness of some tumour cells to chemotherapeutic drugs and provides a rational basis for the use of calmodulin inhibitors in thermochemotherapy.


*Br. J. Cancer* (1986), **53**, 105–114

### Interaction of bleomycin, hyperthermia and a calmodulin inhibitor (trifluoperazine) in mouse tumour cells: II. DNA damage, repair and chromatin changes

P.J. Smith[1], J. Mircheva[2] & N.M. Bleehen[1]

[1]*MRC Unit and University Department of Clinical Oncology and Radiotherapeutics, MRC Centre, Hills Road, Cambridge, CB2 2QH, UK and* [2]*Department of Experimental Therapy of Tumours, Pharmacological Research Institute, Medical Academy, Sofia, Bulgaria.*

**Summary** We have reported in the preceding paper that the treatment of plateau phase mouse EMT6 tumour cells with a combination of hyperthermia (HT; 44°C) and trifluoperazine (TFP; $30\,\mu g\,ml^{-1}$; an inhibitor of calmodulin) greatly enhances the cytotoxicity of the antitumour drug belomycin (BLM). The cytotoxic action of BLM is thought to arise from the induction of DNA damage in a manner which reflects chromatin accessibility. Thus we have studied the effects of the two modifiers (HT and TFP) on chromatin structure and BLM-induced DNA damage. Co-treatment of cells with HT and TFP altered chromatin organisation by the formation and slow resolution of new DNA attachment sites at the nuclear matrix. HT increased drug-induced DNA damage (strand breaks and alkali-labile lesions) by the general depression of repair rather than through the generation of new sites for drug action. TFP produced a more discrete block in the repair of alkali-labile lesions in DNA. Both processes appear to occur for the combination of BLM, HT and TFP, and we propose that the novel chromatin configuration permits the accumulation of potentially lethal DNA strand breaks. Our study indicates the potential value of chromatin/DNA repair modifying regimens for overcoming the poor responsiveness of some tumour cells to chemotherapeutic drugs and provides a rational basis for the use of calmodulin inhibitors in thermochemotherapy.

The anti-tumour glycopeptidic bleomycins are thought to exert their cytotoxic effects by damaging cellular DNA. A number of molecular mechanisms have been proposed (for review see Hecht, 1979) to explain the ability of bleomycin (BLM) to induce the liberation of free bases and the formation of single- and double-strand breaks. A central feature of recent models is the capacity of the antibiotic both to associate with DNA and to complex with ferrous iron. The ferrous oxidase activity of this complex reduces molecular oxygen to the superoxide radical, hydrogen peroxide and the potentially damaging hydroxyl radical (Caspary *et al.*, 1982).

In isolated DNA, BLM has been shown to induce single-strand breaks, alkali-labile sites (representing sites of base loss without cleavage of the phosphodiester bond) and double-strand breaks in the proportion of 5:5:1 (Lloyd *et al.*, 1978). However, various problems are encountered in interpreting the significance of bleomycin-induced DNA damage in intact eukaryotic cells. For example, residual bleomycin may interact with DNA during the preparation of cells for damage analysis (Cox *et al.*, 1974) and the fact that the majority of studies have not distinguished between alkali-labile lesions and true breaks. Furthermore, the complex structure of eukaryotic chromatin restricts the accessibility of BLM such that regions in 'open configuration' (e.g. transcriptionally active regions, possibly associated with the nuclear matrix; Ciejek *et al.*, 1983) are preferentially damaged (Kuo, 1981). Thus, chromatin structure may play an important role in controlling the responses of mammalian cells to BLM by influencing the initial sites and levels of damage, together with the subsequent accessibility of cellular repair enzymes.

The capacity of hyperthermia to potentiate bleomycin cytotoxicity is thought to represent effects at the level of DNA repair (Meyn *et al.*, 1979) perhaps as a consequence of changes in chromatin organisation (Roti Roti, 1982). In the accompanying paper (Mircheva *et al.*, 1986) we have reported that a calmodulin inhibitor (trifluoperazine, TFP) can interact synergistically with hyperthermia (HT) in enhancing BLM toxicity. Calmodulin is a relatively low molecular mass, acidic calcium binding protein which is uniquitous amongst eukaryotic cells. Apart from its apparent importance in the control of cell proliferation (for review see Means *et al.*, 1982) there is recent evidence that calmodulin may have a role in DNA repair given the capacity of calmodulin inhibitors to inhibit the repair of bleomycin induced DNA

Correspondence: P.J. Smith.
Received 15 July 1985; and in revised form 14 October 1985.

© The Macmillan Press Ltd., 1986

damage (Chafouleas *et al.*, 1984) and UV-induced pyrimidine dimers (Charp & Regan, 1985).

This report presents an extension of the above studies. We have investigated the effects of HT and TFP (either alone or in combination) on the DNA repair capacity of BLM treated cells together with changes in chromatin organisation. DNA damage was quantitated by two essentially different techniques which distinguish changes in low levels of DNA damage comprising either true breaks or alkali-labile lesions. Changes in chromatin organisation were studied by determining the accessibility of chromatin to BLM in permeabilized cells and the velocity sedimentation characteristics of nucleoids (deproteinized nuclei) prepared from EMT6 cells.

### Materials and methods

### *Cell culture and treatment protocols*

The maintenance and experimental manipulations of EMT6 (mouse tumour cell monolayer cultures) have been described in the accompanying paper (Mircheva *et al.*, 1986). Freshly prepared, filter-sterilized solutions of $FeSO_4 . 7H_2O$ were the sources of ferrous iron. All experiments were performed on cells maintained in culture for 4 days (i.e. early plateau phase by growth curve analysis).

### *Assays for DNA strand breaks*

*Preparation of cells* Assays were carried out on cultures which had been subjected to a standard protocol for the generation of freeze/thawed (permeabilized) cells directly from monolayer, adapted (Smith, 1984) from the method described by Ganesan *et al.* (1981). Following the treatment of experimental or the sham-treatment of control cultures, monolayers were: washed twice with PBS A, drained well, overlaid with LS buffer (10 mM Tris-HCl, pH 8.0; 100 mM NaCl; 10 mM EDTA; 1 mg ml$^{-1}$ bovine serum albumin), the cells detached by one cycle of freezing and thawing, and cells resuspended by aspiration. The above procedure permitted the assay points in all experiments to be collected within 1 min of the cessation of the treatment. Advantages of this method of analysis are: (a) fast repair events can be analysed; (b) the presence of intracellular EDTA reduces $Ca^{2+}$ and $Mg^{2+}$ dependent endonucleolytic action and prevents the continued action of persistent BLM; and (c) permits the simultaneous analysis of samples from kinetic experiments.

*X-irradiation* Permeabilized (and consequently repair incompetent) cells prepared from monolayer cultures (see below) were irradiated in air as suspensions in cold LS buffer ($5 \times 10^6$ cells ml$^{-1}$ for the generation of X-ray standards for the nucleoid sedimentation assay) at a dose rate of 2.86 Gy min$^{-1}$ (using a 250 Kv, 15 mA, Pantak X-irradiator, Windsor, UK; filtration of 2.32 mm copper half-value thickness).

*DNA undwinding assay* DNA strand breaks were measured by an adaptation of the method described by Kanter and Schwartz (1982) involving the time-dependent partial unwinding of cellular DNA in alkaline solutions. Freeze-thawed cells were resuspended in LS buffer ($5 \times 10^5$ cells ml$^{-1}$) and distributed in 0.5 ml volumes into glass tubes for the determination of unwinding rates in quadruplicate. One set of tubes (P; partial unwinding) were exposed to 0.1 N NaOH (0.5 ml/tube) for 60 min at ice temperature followed by neutralisation with 0.1 N HCl (0.5 ml/tube) and the addition of a detergent/fluorochrome buffer (0.5 ml/tube; the DNA specific dye Hoechst 33342 was used at a final concentration of 0.25 $\mu$M). A second set of tubes (B; maximum unwinding) were handled in a similar way except that the alkaline lysates were sonicated to effect efficient DNA unwinding. A third set of tubes (T; no unwinding) were also handled similarly except that the alkali and acid were premixed (1:1 v/v; 1 ml/tube) before addition. All tubes were homogenised by sonication prior to the measurement of the Hoechst 33342-DNA fluorescence (fluorescence enhancement greater for double than single-stranded DNA) using a Perkin Elmer MPF-4 spectrofluorimeter. Treatment-induced enhancement of DNA unwinding (F; due to strand breaks and alkali-labile damage) was determined by the expression $F = -100 \log[Dx/Dc]$, where Dx and Dc represent the % double-stranded DNA in experimental or control samples respectively (% double-stranded DNA $= 100[P-B/T-B]$; Birnboin & Jevcak, 1981). X-ray calibration of this assay yielded 9.78 F units/Gy (data not shown).

*Nucleoid sedimentation* The method (Cook & Brazell, 1975) detects changes, due to intercalation or DNA strand breakage, in the extent of DNA supercoiling in residual nuclear structures (i.e. nucleoids) obtained by exposure of cells to detergent and high salt conditions. The current version of the technique is essentially that described by Farzaneh *et al.* (1982), adapted as follows. Permeabilized cells were filtered through a 35 $\mu$M monofilament nylon mesh and resuspended in cold PBS A (50 $\mu$l containing $2.5 \times 10^5$ cells) and deposited onto 150 $\mu$l of lysis buffer [giving a final concentration of 2 mM EDTA, 0.5% (v/v) Triton X-100, 100 mM Tris-(hydroxymethyl)-aminomethane pH 8.0 and 1 M NaCl], over 3.8 ml 15–30% linear sucrose gradients containing 1 mM EDTA and 10 mM Tris-(hydroxymethyl)-aminomethane pH 8.0 and 1 M NaCl. Cells were lysed on top of the gradients for 15 or 30 min at room temperature and then centrifuged for 20 min at 12,500 or 25,000 r.p.m. on an MSE Superspeed 65 ultra-centrifuge using a 6 × 4.2 ml swing-out rotor. The sucrose gradients contained 1 $\mu$M Hoechst 33342 (a DNA specific fluorochrome) for the direct determination of the relative distance sedimented (*versus* control) by the nucleoids visualised using near-U.V. illumination. The fluorochrome did not affect nucleoid sedimentation and gave the same results as determining band positions using a spectrophotometer flow cell and measuring absorbance at 254 nm without the attendant pumping artifacts. In titration experiments, ethidium bromide was incorporated into the lysis buffer and the gradient at the concentration specified. Centrifugation conditions (rather than lysis time) were varied to permit either hyperthermia or TFP treated cells to sediment approximately two-thirds of the length of the tube.

To aid the interpretation of the nucleoid experiments Figure 1 shows the various factors which can affect nucleoid sedimentation. Importantly nucleoids can be used to assess levels of DNA strand breaks and changes in chromatin organisation.

**Figure 1** Diagrammatic representation of the proposed structural configurations adopted by de-proteinised DNA loops or domains (attached to a nuclear matrix) in nucleoid preparations. Nuclear matrix attachment sites are indicated by closed triangles, and the DNA domains are normally constrained into a negatively superhelical form (far left). Changes in chromatin organisation can result in: (a) relaxation of supercoiling, (b) increased nucleoid mass due to protein binding, and (c) reduction in domain size due to new attachment sites. DNA damage has various effects: (d) apurinic or apyrimidinic sites (open triangles) cause no loss of supercoiling, (e) single-strand breaks (open circles) give rise to domain relaxation (on the basis of one break per domain) and (f) double-strand breaks result in the loss of domain continuity. Intercalation (eg. by ethidium bromide; EB) causes: (g) relaxation of supercoiling at low EB concentrations, (h) a fully relaxed nucleoid (at 10 $\mu$g EB ml$^{-1}$), and (i) a positively supercoiled nucleoid at high EB concentrations. All of the above changes (except (d) can alter the velocity sedimentation characteristics of nucleoids. Increased loop compactness or mass elevates sedimentation rates, whereas loop relaxation decreases sedimentation rate.

*Chromatin accessibility*

Freeze-thaw permeabilized cells have previously been used to compare chromatin accessibility with respect to exogenously-supplied DNase II (Smith, 1984). Such preparations of EMT6 cells have been used, together with the DNA unwinding assay described above, to monitor chromatin accessibility to the relatively large bleomycin molecule in terms of the capacity of the drug to induce DNA damage under conditions in which the activities of residual nuclear endonuclease(s) or repair enzymes are minimal. Briefly, permeabilised cells (prepared in LS buffer lacking EDTA) were dispensed into glass tubes held on ice (as described above) and BLM added directly into the cell suspension, gently mixed and held for 15 min prior to stopping the reaction by the addition of EDTA (10 mM final concentration). The levels of DNA damage were assayed immediately by the continuation of the DNA unwinding assay as described above.

### Results

*Chromatin changes detected by nucleoid sedimentation*

Roti Roti and Painter (1982) have reported that hyperthermia increases the sedimentation of nucleoids (residual nuclear structures obtained from lysed cells exposed to high salt conditions) by increasing the level of residual protein (Figure 1b). We have used the nucleoid sedimentation technique (see Table I) to demonstrate that a non-toxic hyperthermia treatment (30 min at 44°C) can induce changes in nucleoid sedimentation in EMT6 cells. Surprisingly, TFP (a non-toxic exposure of $30\,\mu g\,ml^{-1}$ for 135 min) also induced a small but invariably positive increase in sedimentation rate.

**Table I** Nucleoid sedimentation characteristics of hyperthermia (HT) and trifluoperazine (TFP) treated EMT6 cells (early plateau phase cultures)

| *Treatment* | *Nucleoid sedimentation (relative distance migrated for various lysis periods)*[a] 15 min | 30 min |
|---|---|---|
| Control | 1.4±0.2 | (set at 1.0) |
| HT[b] | 1.6±0.1 | 1.4±0.1 |
| TFP[c] | 1.9±0.2 | 1.3±0.3 |
| HT+TFP[d] | 4.1±0.6 | 2.7±0.3 |

[a]Mean values (±s.e.) for 2–5 determinations; [b]30 min at 44°C, 90 min at 37°C; [c]$30\,\mu g\,ml^{-1}$ for 135 min; [d]As above with a 15 min pretreatment with TFP prior to hyperthermia.

The combination of HT and TFP was not toxic to EMT6 cells (see Mircheva *et al.*, 1986) under the conditions selected although a greater than additive effect was achieved for the increase in nucleoid sedimentation rate. Indeed, direct comparisons of the relative increase in sedimentation rate induced in HT-treated cells by TFP (i.e. ratio distance sedimented by HT+TFP sample/distance sedimented by HT sample) gave an enhancement ratio of 2.3±1 (6 independent determinations). This effect was found to be long lived (data not shown) in that HT+TFP treated cells showed high sedimentation rates for up to 12 h post treatment incubation at 37°C.

The changes in nucleoid sedimentation for HT+TFP could reflect increases in the negative superhelicity (Cook & Brazell, 1975) of the predominantly deproteinized DNA loops in nucleoids. We have explored this possibility by ethidium bromide titration studies (Figure 1g-i and 2). The concentration of intercalating dye which produces a minimum migration of nucleoids in high salt gradients (representing the relaxed state of supercoils; Figure 1h) is a measure of the average superhelical density. The minimum sedimentation concentration was found to be the same for control, HT, TFP or HT+TFP treated cells. Thus the changes in nucleoid sedimentation probably reflect increased nucleoid mass or DNA loop compactness.

**Figure 2** Change in sedimentation rate of EMT6 nucleoids as a function of ethidium bromide concentration within sucrose gradients. (○) control; (●) hyperthermia; (△) TFP; (▲) hyperthermia+TFP. Data points represent arithmetic means (range ~±5%) of two determinations. See **Table I** for treatment protocols.

The nucleoid sedimentation technique can be used to infer the presence of DNA strand-breaks by the measurement of the relaxation of DNA supercoiling (Cook & Brazell, 1975). Conversely, the assay can be calibrated against an agent which induces random DNA strand-breaks (e.g. X-radiation; Figure 1e) throughout the genome as a means of quantitating the average size of DNA domains.

Figure 3 shows the X-ray dose dependence of the decrease in nucleoid sedimentation rates for control, HT, TFP and HT+TFP treated cells (using repair incompetent, permeabilized preparations). The results for control cells are similar to those reported for HeLa nucleoids (Cook & Brazell, 1975). The treatment of EMT6 cells with either TFP or HT did not appear to modify X-ray responsiveness. However, there is clear evidence that cells treated with the combination (HT+TFP) are less responsive to X-ray induced breaks for doses above 1 Gy. We conclude that the combination treatment results in an approximately two-fold decrease (compare 1 Gy and 2 Gy data points; Figure 3) in the average size of DNA supercoiled domains.

**Figure 3** Change in sedimentation rate of EMT6 nucleoids as a function of X-ray dose for samples of permeabilized cells irradiated immediately prior to nucleoid preparation. Data represent arithmetic means (±s.e.) of values from 4–6 experiments. See **Table I** for cell treatment protocols. (○) control; (●) hyperthermia; (△) TFP; (●), combined hyperthermia and TFP treatment.

*Establishment of treatment protocol for DNA damage/repair studies*

Preliminary studies resulted in the adoption of the treatment protocol (in which cells are treated with HT, TFP and BLM simultaneously) described by Mircheva *et al.* (1986). The protocol was based, in part, on a series of observations and deductions, including:

(i) Hyperthermia alone causes long-lived DNA strand breaks to appear in cellular DNA during and soon after heat exposure. A 30 min exposure at 44°C resulted in a low level of breakage which did not alter significantly upon incubation of cells at 37°C (Figure 4).

(ii) BLM-treated EMT6 cells rapidly establish a plateau level of DNA damage (Figure 5a) which is dose dependent up to 20–40 μg ml (compare Figure 5a and 5b).

(iii) Enhancement of BLM activity by changes in $Fe^{2+}$ supply has a transient effect upon levels of damage which eventually return to the normal steady-state level. (Figure 5a).

(vi) A 40 μg BLM $ml^{-1}$ treatment results in a steady level of DNA damage maintained over a 2 h period (Figure 5b) and simultaneous treatment with HT (44°C for the first 30 min of drug exposure) results in a time dependent increase in DNA damage (Figure 5b).

(v) Pretreatment of cells with HT prior to BLM exposure was less effective than simultaneous

**Figure 4** Appearance of DNA strand breaks (or alkali-labile lesions) in hyperthermia treated EMT6 cells as a function of incubation period at 37°C. DNA strand breakage indicated by the decrease in DNA double-strandedness for a standard period of alkaline denaturation (see **Materials and methods**). (○) 30 min at 44°C; (●) 60 min at 44°C.

**Figure 5** Kinetics of induction of DNA damage, for a continuous exposure to BLM, monitored by the alkaline denaturation assay (see **Materials and methods**). (a): (○) 5 $\mu$g BLM ml$^{-1}$; (△) 10 $\mu$g BLM ml$^{-1}$; (◇) 20 $\mu$g BLM ml$^{-1}$; (▲) 20 $\mu$g BLM ml$^{-1}$ supplemented with 3 $\mu$M $Fe^{2+}$. (b): (○) 40 $\mu$g BLM ml$^{-1}$ alone; (●) 40 $\mu$g BLM ml$^{-1}$ with an initial 30 min exposure at 44°C.

treatments in enhancing levels of DNA damage (data not shown).

(vi) Removal of BLM results in the rapid disappearance of DNA damage ($T_{1/2}=9$ min; data not shown).

(vii) Treatment of permeabilized cells at 0–4°C (i.e. repair incompetent cells; see **Materials and methods**) with BLM reveals that in the absence of repair only 15±5% of total lesions represent true breaks, the remainder being alkali-labile lesions (data not shown).

Together with evidence in the literature (Lloyd *et al.*, 1978), our observations suggest that many of the initial lesions induced in cellular DNA are alkali-labile sites. Repair of such sites involves cleavage by an endonuclease (to yield true breaks) followed ultimately by a ligation event. Thus the steady-state level of damage could reflect a mixture of initial lesions and repair intermediates. Hyperthermia (Figure 5b) could act to modify the balance between induction and repair resulting in a time dependent increase in total lesions.

Changes in the steady-state level of DNA due to HT or TFP exposure should be a sensitive indicator of cellular repair rates. Interpretation of such changes is provided by the use of the alkaline-denaturation assay (detecting all lesions) and the neutral nucleoid sedimentation assay which should selectively monitor true breaks (incorporating repair intermediates; Figure 1e and f).

### *BLM-induced DNA damage in HT and TFP treated cells*

Based upon the rationale provided above and adopting the treatment schedule outlined in the preceding paper, we have compared the results of the alkaline-denaturation and the nucleoid sedimentation assays (Table II). Lesion frequencies were determined by expressing DNA strand breakage events in terms of the X-ray dose required to produce an equivalent effect by comparison with calibration standards (see **Materials and methods** and Figure 3), and calculating the corresponding number of strand breaks using the factors given in the footnote to the Table II. A direct comparison of the two assays for the levels of DNA damage induced in control cells suggests that there is no significant level of alkali-labile sites maintained under the prevailing steady-state conditions. Considering the results of the alkaline-denaturation assay, both HT and TFP treatments significantly increase the number of lesions. TFP appears to be

**Table II** Effects of hyperthermia (HT) and trifluoperazine (TFP) on bleomycin (BLM)-induced DNA damage in EMT6 cells

| | *BLM-induced (40 µg ml$^{-1}$ x 2 h) DNA damage detected by:* | | | |
|---|---|---|---|---|
| | *Alkaline denaturation*[b] | | *Nucleoid sedimentation*[c] | |
| *Treatment*[a] | *F*[d] | *Lesions per 10$^{10}$ daltons*[e] | *RDM*[f] | *lesions per 10$^{10}$ daltons*[e] |
| Control | 10.3±0.3[g] | 3.0 | 0.38±0.05 | 3.4 |
| HT | 21.7±0.6 | 5.9 | 0.33±0.04 | 4.8 |
| TFP | 34.3±1.7 | 9.5 | 0.49±0.02 | 2.4 |
| HT+TFP | 48.3±1.6 | 13.2 | 0.47±0.03 | 5.7 |

[a]See **Table I** for treatment protocols; [b]Detects strand breaks including those arising from alkali-labile lesions; [c]Detects frank breaks alone; [d]$F=[-100\log(Dx/Dc)]$; See **Materials and methods**; [e]Calculated with reference to X-ray standards, assuming: X-rays induce 2.7 breaks/10$^{10}$ daltons mol.wt. DNA/Gy (Kohn *et al.*, 1976), and 28% of breaks arise from alkali-labile lesions (Lennartz *et al.*, 1973); [f]RDM = relative distance migrated (with respect to treated cells not exposed to BLM); [g]Values represent arithmetic means (±s.e.) for 2–6 experiments.

particularly effective, yielding a greater than 3 fold increase in lesion frequency. The combination of HT and TFP results in a somewhat less than additive effect. The nucleoid sedimentation assay indicates only a marginal increase, 1.2-fold) in true breaks for HT treated-cells whereas in the case of TFP treated cells there is a significant decrease (30%; $P<0.05$) in the level of true breaks. This finding is in direct contrast to the results of the alkaline-denaturation assay. The combination of HT+TFP moderately increases the level of true breaks (1.7-fold greater than control) although the increase in lesion frequency is considerably less than the 4.4-fold elevation observed under alkaline-denaturation.

### *Accessibility of chromatin to BLM in hyperthermia treated cells*

We have attempted to ask the direct question of whether heat-induced changes in protein/DNA interactions result in new sites on DNA for BLM attack, giving rise to the observed increase in the frequency of drug damage (see Table II).). For this purpose, permeabilized cells from HT-treated cultures were exposed to low levels of bleomycin at ice temperature, such that the induction of DNA damage reflects accumulated BLM-DNA interactions without repair involvement (Figure 6). For both 30 min and 60 min HT treatments there were fewer DNA sites accessible to BLM molecules in heated cells compared with controls.

We conclude that although the relatively large antibiotic (mol.wt > 1400) has restricted access to DNA within chromatin of HT-treated cells there is a general elevation in the frequency of DNA lesions (Figure 5b; Table II) in intact cells due to a decreased cellular repair capacity resulting in an accumulation of alkali-labile sites and relatively long-lived true breaks.

### Discussion

The present study follows our previous observations (Mircheva *et al.*, 1986) that HT and TFP can interact in EMT6 cells to produce a significant enhancement of bleomycin cytotoxicity. We have investigated the role of changes in chromatin structure together with the concomitant effects on the repair of BLM-induced DNA damage. We

**Figure 6** Effect of moderate (left panel) or extensive (right panel) hyperthermia treatment on the sensitivity of DNA in permeabilized EMT6 cells to breakage by BLM exposure (15 min at ice temperature). Data points in (a) represent means (±s.e.) for quadruplicate determinations. Data in (b) derived from 3 experiments, each point represents the mean (±s.e.) of quadruplicate determinations. Moderate hyperthermia was 30 min at 44°C followed by 90 min at 37°C. Extensive hyperthermia was 60 min at 44°C followed by 60 min at 37°C. (○) control (●) hyperthermia.

provide evidence that when cells are treated with a combination of HT and TFP there are significant changes in the interaction of DNA with its protein matrix and we propose that such changes greatly enhance the probability of lethal damage being expressed in BLM-treated cells due to a depression of DNA repair functions.

Our findings relate to three specific areas, *viz.* (i) the effects of HT and TFP (the action of which is interpreted in terms of its effect on calmodulin-dependent processes) on higher orders of chromatin structure, (ii) the effects of HT and TFP on the induction and repair of BLM-induced DNA damage, and (iii) the dependence of DNA repair functions on chromatin organisation in handling biologically relevant DNA damage.

We have used the nucleoid sedimentation technique (Figure 1) to reveal changes in chromatin structure and also as a method of detecting DNA strand breaks. Nucleoids are residual nuclear complexes of supercoiled DNA associated with a non-histone protein matrix (resistant to dissociation by high salt/detergent treatment). The sedimentation rate of nucleoids in neutral sucrose gradients is determined by nucleoid mass and the compactness of the DNA loops or domains (domain size being a function of the frequency of matrix attachment sites). Under normal conditions the DNA in nucleoids is negatively supercoiled and relaxation (decreasing compactness) of domains can be effected by either the presence of DNA strand breaks or by the generation of an opposing positive twist due to the intercalation of molecules (such as ethidium bromide) between the base pairs. Nucleoid compactness can also be affected by changes in the average domain size resulting from the formation or loss of matrix attachment sites.

We found that the treatment of EMT6 cells with HT and TFP (either alone or in combination) did not greatly alter the average superhelicity of DNA domains although the absolute sedimentation rates of HT and HT+TFP treated nucleoids were increased. In the case of HT the sedimentation change is known to relate to an increase in nucleoid mass (Roti Roti & Painter, 1982; Figure 1b). The significant increase in sedimentation rate for HT+TFP treated cells was suggestive of a further increase in DNA compactness. Thus, X-irradiation was used as a means of determining average domain size in the nucleoid preparations (Cook & Brazell, 1975; Lipetz *et al.*, 1982). X-irradiation of control cells at a dose of 1.9 Gy reduces the number of intact domains to 37% (i.e. $1/e \times 100$) of the original and this corresponds, assuming 1.94 true strand breaks/$10^{10}$ daltons/Gy (see footnote to Table II and **Results**), to an average domain size of $2.7 \times 10^9$ daltons mol.wt DNA. This estimation of domain size is comparable with previous estimates of $1.8 \times 10^9$ daltons for human dermal fibroblasts and $3.8 \times 10^9$ daltons for rat spleen lymphocytes (for review see Lipetz *et al.*, 1982). HT or TFP alone did not produce a significant effect on domain size, whereas there was a greater than two-fold decrease in domain size for cells treated with the combination of agents.

We propose that the change in domain size reflects the formation of new attachment sites at the protein matrix due to two factors: (i) the increased availability of cellular and nuclear proteins for interaction with DNA in HT-treated cells (Roti Roti, 1982) and (ii) a significant role for calmodulin in regulating the association and dissociation of DNA at attachment sites. Thus HT freezes (by protein binding) new attachment sites (i.e. a combination of conditions b and c in Figure 1) formed in cells with inhibited calmodulin activity. The effect is not observed in cells treated with TFP alone since such new attachment sites are normally transient and involve only a minority of domains at a given time. Indeed it is interesting to speculate that heat shock may be a useful method for revealing dynamic changes in domain reorganisation.

In attempting to interpret the results of DNA damage/repair experiments we have determined whether the enhancement of the DNA-damaging potential of BLM was related to the availability of new sites within chromatin for drug attack. BLM was used as a direct probe for chromatin accessibility in permeabilized (repair incompetent) EMT6 cells. We found that HT treatment (even for non-toxic exposures of 30 min at 44°C) restricts the access of active drug to DNA. This observation is consistent with the notion that an increase in nuclear protein in HT-treated cells reduces the accessibility of DNA (Roti Roti, 1982) and the observation (Braun & Hahn, 1975) that rodent cells heated at 43°C retain less BLM than controls exposed at 37°C. Thus, we conclude that the increase in drug-induced DNA damage in HT-treated cells probably represents an underestimation of the predominent effect of HT on reducing cellular DNA repair capacity.

It is known that BLM can induce a significant proportion of alkali-labile lesions (e.g. apyrimidinic or apurinic sites; i.e. AP sites; Lloyd *et al.*, 1978) and our preliminary experiments (noted in **Results**) on the drug-induced DNA damage in repair incompetent EMT6 cells suggest that AP sites may be initially the predominant class of lesions. However our kinetic studies indicate that repair limits the induction of DNA damage to such an extent that no AP sites can be detected under steady-state conditions, presumably due to the optimal cleavage of such lesions to repair intermediates (i.e. strand breaks). Thus the use of putative inhibitors (such as TFP; Chafouleas *et al.*, 1984) after BLM removal would only monitor effects on a restricted class of lesions. Consequently we have adopted the approach that changes in the levels of BLM-induced DNA damage for simultaneous exposures to HT and TFP, effectively monitor the capacity of rapair processes to limit lesion accumulation. The effect of HT appears to be a general depression in the repair of AP sites and the resolution of strand breaks. On the other hand, TFP appears to have a more discrete effect on the repair incision events at AP sites rather than ligation (although there are presumably fewer cleaved AP sites for processing by the ligation system). The effects of HT and TFP in combination at the level of DNA repair result in an unexpected (given the action of the single agents) increase in the accumulation of frank breaks. Given the possible role of calmodulin in controlling dynamic changes in domain structure it appears that changes in domain structure may be prerequisite for the appropriate action of cellular repair enzymes.

Taking the data overall BLM cytotoxicity can be related to DNA damage in terms of the steady-state level of strand breaks which reflects the balance between repair and the induction of damage. A given steady-state level of strand breaks fixes the probability of inducing potentially lethal lesions (such as double strand breaks representing coincident lesions or the overlap of repair incision events; Soniger *et al.*, 1982). Consistent with this model, the accumulation of AP sites (in the presence of TFP) is not a particularly lethal process (Mircheva *et al.*, 1986) whereas the enhancement in DNA strand breakage by the combination of HT and TFP is highly toxic to drug treated cells.

The main conclusions are: (i) Calmodulin participates in the control of transitions in chromatin domain structure, (ii) HT modifies chromatin structures to reduce accessibility for a DNA interactive drug although the cell accumulates abnormally high levels of damage due to a prevailing general depression in repair, and (iii) HT and a calmodulin inhibitor can interact in a non-toxic manner to produce novel changes in chromatin structure which favour the formation of predominantly lethal DNA damage.

At the clinical level our studies provide a theoretical rationale for combining HT and calmodulin inhibitors in modifying the innate responsiveness of resistant tumour cells. Since chromatin structure varies according to cell-cycle age and differentiation status, we suggest that such combined modalities may provide some degree of cell type specificity for drug cytotoxicity.

The authors thank Ms C.O. Anderson for technical assistance. J.M. was in receipt of a visiting fellowship of the International Atomic Energy Agency.

### References

BIRNBOIN, H.C. & JEVCAK, J.J. (1981). Fluorometric method for the rapid detection of DNA strand breaks in human white blood cells produced by low doses of radiation. *Cancer Res.*, **41**, 1889.

BRAUN, J. & HAHN, G.M. (1975). Enhanced cell killing by bleomycin and 43°C hyperthermia and the inhibition of recovery from potentially lethal damage. *Cancer Res.*, **35**, 2921.

CASPARY, W.J., LANZO, D.A. & NIZAK, C. (1982). Effect of deoxyribonucleic acid on the production of reduced oxygen by bleomycin and iron. *Biochemistry*, **21**, 334.

CHAFOULEAS, J.G., BOLTON, W.E. & MEANS, A.R. (1984). Potentiation of bleomycin lethality by anticalmodulin drugs: A role for calmodulin in DNA repair. *Science*, **224**, 1346.

CHARP, P.A. & REGAN, J.D. (1985). Inhibition of DNA repair by trifluoperazine. *Biochim. Biophys. Acta*, **824**, 34.

CIEJEK, E.M., TSAI, M.-J. & O'MALLEY, B.W. (1983). Actively transcribed genes are associated with the nuclear matrix. *Nature*, **306**, 607.

COOK, P.R. & BRAZELL, I.A. (1975). Supercoils in human DNA. *J. Cell. Sci.*, **19**, 261.

COX, R., DAOUD, A.H. & IRVING, C.C. (1974). Damage of rat liver deoxyribonucleic acid by bleomycin. *Biochem. Pharmac.*, **23**, 3147.

FARZANEH, F., ZALIR, R., BRILL, D. & SHALL, S. (1982). DNA strand breaks and ADP-ribosyl transferase activation during cell differentiation. *Nature*, **300**, 363.

GANESAN, A.K., SMITH, C.A. & VAN ZEELAND, A.A. (1981). Measurement of the pyrimidine dimer content of DNA in permeabilized bacterial or mammalian cells with endonuclease V of bacteriophage $T_4$. In *DNA Repair: A Laboratory Manual of Research Techniques*, Friedberg, E.C. and Hanawalt, P.C. (eds) Marcel Dekker: New York.

HECHT, S.M. (ed), (1979). *Bleomycin: Biochemical and Biological Aspects*. Springer-Verlag: New York.

KANTER, P.M. & SCHWARTZ, H.S. (1982). A fluorescence enhancement assay for cellular DNA damage. *Molec. Pharmac.*, **22**, 145.

KOHN, K.W., ERIKSON, L.C., EWIG, R.A.G. & FRIEDMAN, C.A. (1976). Fractionation of DNA from mammalian cells by alkaline elution. *Biochemistry*, **15**, 4628.

KUO, M.-T. (1981). Preferential damage of active chromatin by bleomycin. *Cancer Res.*, **41**, 2439.

LENNARTZ, M., COQUERELLE, T., BOPP, A. & HAGEN, U. (1973). Effect of oxygen on DNA strand breaks in irradiated thymocytes. *Int. J. Radiat. Biol.*, **24,** 621.

LIPETZ, P.D., GALSKY, A.G. & STEPHENS, R.E. (1982). Relationship of DNA tertiary and quarternary structure to carcinogenic processes. *Adv. Cancer Res.*, **36,** 165.

LLOYDS, R.S., HAIDLE, C.W. & HEWITT, R.R. (1978). Bleomycin-induced alkaline-labile damage and direct strand breakage of PM2 DNA. *Cancer Res.*, **38,** 3191.

MEANS, A.R., CHAFOULEAS, J.A., LAGACE, L., LAI, E. & STEIN, J.P. (1982). Multiple roles for calmodulin in the regulation of eukaryotic cell metabolism. In *Gene Regulation,* p. 307. Academic Press: New York.

MEYN, R.E., CORRY, P.M., FLETCHER, S.G. & DEMETRIADES, M. (1979). Thermal enhancement of DNA strand breakage in mammalian cells treated with bleomycin. *Int. J. Radiat. Oncol. Biol. Phys.*, **5,** 1487.

ROTI ROTI, J.L. (1982). Heat-induced cell death and radiosensitization: Molecular mechanisms. *Natl Cancer Inst. Monogr.*, **61,** 3.

ROTI ROTI, J.L. & PAINTER, R.B. (1982). Effects of hyperthermia on the sedimentation of nucleoids from HeLa cells in sucrose gradients. *Radiat. Res.*, **89,** 166.

MIRCHEVA, J., SMITH, P.J. & BLEEHEN, N.M. (1986). Interaction of bleomycin, hyperthermia and a calmodulin inhibitor (trifluoperazine) in mouse tumour cells: I *in vitro* cytotoxicity. *Br. J. Cancer,* **53,** 99.

SMITH, P.J. (1984). Relationship between a chromatin anomaly in ataxia telangiectasia cells and enhanced sensitivity to DNA damage. *Carcinogenesis,* **5,** 1345.

SONIGER, M.A., HITTLEMAN, W.N. & POLLARD, M. (1982). The relationship between DNA and chromosome damage after bleomycin treatment: Dose-response measurements. *Mutat. Res.*, **93,** 249.